# Insights from coronial recommendations for preventing natural deaths in sport and recreation in Québec, Canada

**DOI:** 10.3389/fpubh.2024.1389675

**Published:** 2024-07-31

**Authors:** Philippe Richard, Paul-André Perron, Jérémie Sylvain-Morneau, Paul Poirier

**Affiliations:** ^1^Direction de la sécurité dans le loisir et le sport, Ministère de l’Éducation, Québec, QC, Canada; ^2^Bureau du coroner du Québec, Québec, QC, Canada; ^3^Institut national de santé publique du Québec, Québec, QC, Canada; ^4^Institut universitaire de cardiologie et de pneumologie de Québec, Université Laval, Québec, QC, Canada; ^5^Faculty of Pharmacy, Université Laval, Québec, QC, Canada

**Keywords:** sport and recreation, mortality, natural causes, sudden cardiac death, coronial recommendations, automated external defibrillators

## Abstract

**Introduction:**

This descriptive retrospective study analyzed coronial recommendations for natural deaths in sport and recreation from January 2006 to December 2019 using data from the Bureau du coroner du Québec.

**Methods:**

Reports with recommendations were analyzed by sex, age group, cause of death, context, and activity. The nature of recommendations was assessed using a public health-based model. Thematic analysis was conducted following a four-phase approach in which themes developed were emphasized and further connected with existing literature.

**Results:**

Reports involving individuals aged 18–24 and reports related to ice hockey were significantly more likely to contain recommendations. Reports related to individuals ≥45 years old, or related to cycling or hunting had higher death frequencies, but relatively low recommendation rates. Most recommendations aligned with the public health-based model but specifying implementation time frames was rare (11.7%). Nearly 60% of coroner’s recommendations focused on automated external defibrillator implementation, delivery and training.

**Discussion:**

Mitigation of sudden cardiac arrest risk for individuals ≥45 years old, timely treatment of life-threatening arrhythmias especially for activity practiced in remote regions and specifying implementation time frames were identified as improvement areas. The multi-faceted approach to enhancing public access defibrillation developed by the International Liaison Committee on Resuscitation in 2022 addresses recurrent themes covered by coroners and holds the potential to inform evidence-based decision making.

## 1 Introduction

Understanding and preventing fatalities in the context of sport and recreation activities necessitates a comprehensive analysis of contributing factors (1, 2). Surveillance studies play a pivotal role in providing essential data regarding fatal outcomes associated with these activities, which, in turn, inform safety practices (3, 4). Similarly, exploring coronial recommendations stemming from death investigation reports helps identify priority areas of action and evidence-based prevention strategies while maximizing the coroners’ contribution to death prevention (1, 5–7).

In the province of Québec, Canada, between January 2006 and December 2019, 297 sport and recreation-related deaths resulted from natural causes (4). While the examination of trends in death frequency, rates, and etiology is necessary (4, 8, 9), investigating coronial recommendations is crucial for holistically documenting this public health issue as part of an integrated management approach (1, 5–7).

Under the Coroners Act (CQLR, chapter C-68.01, article 3), Québec’s coroners have the legal authority to issue official recommendations aimed at enhancing the protection of human life in their investigation reports: “*If pertinent, the coroner may also, at an investigation or an inquest, make any recommendation directed toward better protection of human life*” (4, 10). These recommendations are of vital significance, as they appear in only approximately 4% of all coronial reports in Québec (all mortality causes) and may serve as a template to preventive measures aimed at protecting human life and preventing deaths under similar circumstances. A recommendation is formally defined as a textual statement within the Recommendations section of an investigation report, comprising a warning, advice, caution, alert, or a suggested action aimed at preventing future fatalities (5).

Québec’s coroner are trained in applying public health principles during investigation and recommendation development (11), thereby optimizing potential preventive approaches (5, 6). The extent to which the actual sport and recreation-related recommendations align with scientific principles of injury prevention remains, nevertheless, undocumented. In addition, recipients (organizations responsible for implementing a given recommendation, as identified by the coroner) of recommendations in Québec are not legally obligated to implement them, and non-adherence occurs (12). Since November 2022, however, recipients are required to confirm that they have considered the recommendations and inform the Québec Chief Coroner of their intended corrective measures (10). The situation in the province of Québec aligns with Australia, where mandatory responses to coronial recommendations provided no guarantee of their implementation (7). In this context, conducting a thematic analysis of clustered recommendations related to natural deaths associated with sport and recreation can help emphasize recurring themes as well as their scientific and medical significance (1, 13), thereby uncovering significant information that might have otherwise been overlooked.

This study, therefore, explored coronial recommendations related to natural deaths in the context of sport and recreation in Québec for the period between January 2006 and December 2019. It investigated demographic and contextual specifics of cases, the nature of recommendations according to public health principles as well as the addressed themes.

## 2 Methods

### 2.1 Design and data source

Expanding on prior investigations (4), this descriptive retrospective study used data extracted from the computerized database of the Bureau du coroner du Québec (CD-BCQ). This repository provides standardized data for each instance of mortality, utilizing a codification system rooted in the International Classification of Diseases (ICD-10). All sport and recreation cases for the period between January 2006 and December 2019 (*n* = 2,234) were previously identified using algorithms based on preselected keywords and ICD-10 codes (4), procedures that specifically targeted cases that coroners coded as “while playing a sport” or “while participating in a game or leisure activities,” as well as a meticulous screening process depicted in the data flow diagram of a previous investigation (4). A dual data entry process was applied to all fatalities related to sport and recreation in which investigators delineated a spectrum of factors including the activity associated with each death as well as contextual information, following the detailed description outlined in the Data Processing section of an earlier research (4). Basic information related to the etiology of the deaths was extracted from the coroners’ reports. The specific causes of cardiac death recorded in some coroner reports (namely myocardial infarction, malignant cardiac arrhythmia, or aortic lesions) and the broad diagnostic category (e.g., “natural death of cardiac origin”) were categorized as cardiac deaths. Deaths that did not meet these criteria were categorized as noncardiac. General information was extracted directly from the CD-BCQ: age at death, sex, year of death and presence, *n* ≥ 1 of a recommendation.

Among this sport and recreation-related deaths dataset (*n* = 2,234), the 1937 fatalities related to unintentional injury were excluded based on their ICD-10 codes (all unintentional injuries codes: V01-X59) (4), yielding 297 deaths of natural causes (as concluded in the textual component of the reports). Among these cases of natural death associated with sport and recreation, the current investigation focused on the reports that included one or multiple recommendations. Cases marked as “Yes” in the recommendation’s variable of the CD-BCQ, in which the presence of recommendations was confirmed in the Recommendations section of the report (100%), were identified (*n* = 26). The research did not require ethical approval as it relied exclusively on publicly available information. Under the *Coroners Act* (CQLR, chapter C-68.01), coroners’ reports are public records (4, 10).

### 2.2 Data processing

A recommendation was defined as a formal textual statement, within the Recommendations section of the investigation report, that comprised warning, advice, caution, alert, or a suggested action aimed at preventing future fatalities (5). The term ‘recommendations’ was employed in analogous studies conducted by other research groups analyzing coroners’ data related to recommendations (1, 5–7). All recommendations (*n* = 60) were extracted from the 26 investigation reports and were logged in Microsoft Excel and Microsoft Word. The reports contained only unique coronial recommendations (multiple associated cases can sometimes feature the exact same recommendations (1), which was not the case for the current study), and they were hence all analyzed (*n* = 60).

A method based on globally recognized and extensively applied public health conceptual models related to causes and prevention of injuries was employed to quantify and depict the nature of the coroners’ recommendations (5, 6). Two investigators (PR and PAP) separately examined and categorized each recommendation based on the seven components of the model (the investigators analyzed the first five recommendations together to promote a common understanding of the process). In the case of the risk/contributing factor component (results section), the investigators examined relevant information within all sections of the investigation report. This is because coroners in Québec are specifically instructed to extensively discuss these aspects within the reports but not to textually include the risk/contributing factors directly in the formal textual statements of the recommendation (11). Since coroners’ reports are public records in Québec (10), they are provided to the recipients in their entirety along with the recommendations, and the risk/contributing factors are thoroughly discussed within the Analysis section of the reports. Discrepancies in data entry between the two investigators were resolved through discussion and level of agreements was recorded (presented in the Results section). A board-certified cardiologist (PP) was assigned to intervene in addressing any persistent discrepancies, which was not needed. The results of this analysis, along with all recommendations, were sent to this expert for final validation.

Analysis of recommendations-related themes was inspired by two very similar methods, one involving a four-phase approach (13) and the other consisting of six phases (14). Although both models helped researchers gain insight into qualitative data analysis and influenced the subsequent process, the former model (13) was adopted to describe the different stages of the analysis in the current study. Accordingly, during the initialization phase of the analysis, PR and PAP individually read all recommendations in French at least three times, took notes, and searched for recurring themes and abstractions using a color-based conceptual coding system, (the investigators collectively analyzed the initial five recommendations to foster a shared comprehension of the procedure). In the construction phase, clusters of analogous codes were compared and further revised. Labels were formulated using meaningful sentences and assigned to each cluster, respecting the principle of mutual exclusiveness (13). These themes were then placed in a table by each investigator independently to promote a holistic appreciation of the developed items. The rectification phase consisted in the individual revision and testing of themes in relation with each other and with the initial recommendations. A meeting was then conducted to compare themes and their interpretations between investigators. Similarities, discrepancies, connections and relationships with the study topic were thoroughly discussed. On the nine themes individually formulated by each investigator, seven were identical in meaning while two necessitated a discussion to detail the meaning and applicability. A common matrix was elaborated based on the common understanding and wording of the themes formulated (*n* = 9) and was validated by the cardiologist investigator (PP). This expert also sent topic-related scientific articles to the two investigators to sharpen and guide their reflections in preparation for the finalization phase of the analysis. Based on the validated matrix, PP and PAP proceeded to quantifying independently the themes (presence or absence) for each recommendation. Discrepancies were discussed between the two investigators until consensus was reached and level of agreements was recorded. During the finalization phase of the thematic analysis, the authors further linked the themes developed in this study with existing literature and with the specificities of the situation in the province of Québec. The result of this process is reported in the Discussion section of the manuscript. For this article, the matrix was translated by the linguistic services of the Ministère de l’Éducation du Québec. The final wording and meaning of translated themes were validated by the three investigators implicated in the elaboration of the matrix.

Agreement levels for both the analysis of nature and themes were reported (first five recommendations excluded) as prevalence-adjusted bias-adjusted kappa (PABAK) coefficients. PABAK coefficients were preferred over non-adjusted Kappa coefficients to assess inter-rater reliability because, in the present setting, the two classes used by the raters (“Presence” and “Absence”) are highly imbalanced: the maximum absolute value of the Prevalence Index (PI) is 1.00 for the components related to the nature of coroner’s recommendations and 0.96 for the themes (15).

### 2.3 Data analysis

Participation and population-based rates were calculated for all cases that were associated with a recommendation. Denominators for participation-based rates were obtained from the 2009–2010 (16) and 2015–2016 (17) editions of the *Étude des blessures subies au cours de la pratique d’activités récréatives et sportives au Québec* (ÉBARS), and exclusively included the 24 activities common to both ÉBARS editions, as previously reported (4). The count of deaths was divided by the annual participation figures from each study (ÉBARS), resulting in a rate per 100,000 participant-years. Population-based rates were derived from age and sex estimates for Québec’s administrative regions (*n* = 17) as of July 1 of each year, obtained from the Institut de la statistique du Québec’s website (18). Population-based rates, presented per 100,000 person-years, reflect the annual death count relative to the corresponding year’s population. Since the number of deaths with recommendations is very low (only 20 with the ÉBARS denominator and 26 with the populational denominator), only crude rates were used. In a previous study where the same denominators were applied for unintentional injury deaths (the number of deaths was higher, *n* = 1957), high concordance was found between crude and age (≤ 17, 18–44, 45–64, ≥ 65 years) and sex-adjusted annual incidence rates and only crude rates were presented (4, 19). Confidence intervals were calculated at a 95% significance level using the Gamma distribution method (20). In accordance with Statistics Canada guidelines, estimates with coefficients of variation (CV) > 33.3% are categorized as unreliable, and estimates with CVs ranging from 16.6 to 33.3% require careful interpretation (16, 17). Poisson regression was applied to examine shifts in recommendations’ rates over the 14-year period, utilizing incidence rate ratios (IRRs). Due to the low number of deaths with recommendations (*n* = 26 and *n* = 20), IRRs were not stratified by activity. The analysis involved the assessment of annual rate variations between consecutive years using linear trend analysis, with a significance level set at 0.05 (19).

The frequency of recommendations as well as components related to the nature of recommendations and themes were determined for individual activities and for all activities clustered using descriptive statistics (1, 5, 6). Age groups ≤5, 6–11, and 12–17 years were combined due to a low number of cases. Activities with less than five deaths were included in the category “other activities.” Significant differences between the recommendation and non-recommendation groups were examined using Fisher’s exact tests, which were preferred over chi-square tests due to the low number of deaths and recommendations (6, 21). To control for the global type-1 error rate, global tests were conducted for each variable (sex, age, cause of death, context, and activity) (5). If the test yielded a statistically significant result, the different variable-related modalities were compared within their respective group (e.g., ≤ 17 years old vs. all other age groups combined) (1).

SAS software (©2019–2020, SAS Institute Inc., Cary, NC, United States) was employed for the analysis. To assess the variance in participation-based rates, the working assumption was that denominator values remained stable as the primary source of variability typically originated from the numerator due to its lower values in most cases (19).

## 3 Results

### 3.1 Frequency and rate of coroners’ recommendations

Over the 14-year period studied, there were 26 cases of death of natural causes associated with sport and recreation that contained minimally a recommendation (1.86 per year), yielding a population-based rate of 0.02 per 100,000 person-years (95% CI: 0.02–0.03; CV: 19.6%). Considering exclusively the at-risk population in the ÉBARS and the 24 activities matching in both editions of this survey, 20 reports with at least a recommendation were numbered and the participation-based rate was 0.02 per 100,000 participant-years (95% CI: 0.01–0.04; CV: 22.4%). Significant average yearly decreases in the population-based (IRR 0.88; 95% CI, 0.80–0.98; *p* = 0.0149) and the participation-based (IRR 0.88; 95% CI, 0.78–0.99; *p* = 0.0271) recommendation rates were observed over the 14-year period.

Table 1 presents a descriptive statistical overview of the frequency and proportion of cases involving coroners’ recommendations in comparison to cases without recommendations, categorized by the deceased’s sex, age group, cause of death, and the context and activity at the time of death.

**Table 1:** Frequency and rate of coroners’ recommendations for natural deaths associated with sport and recreation, by sex, age group and activity in Québec, Canada, from January 1, 2006, to December 31, 2019 (inclusive).

	No recommendation (%)	Recommendation (%)	Total (%)	*P*-value	Rate: percentage of reports containing at least one recommendation
Sex					
Female	20 (7.4)	2 (7.7)	22 (7.4)	1.0000	9.1
Male	251 (92.6)	24 (92.3)	275 (92.6)		8.7
Age group (years)					
≤ 17	9 (3.3)	3 (11.5)	12 (4)	0.0771	25
18–24	9 (3.3)	4 (15.4)	13 (4.4)	0.0193^a^	30.8
25–34	15 (5.5)	1 (3.8)	16 (5.4)	1.0000	6.3
35–44	39 (14.4)	1 (3.8)	40 (13.5)	0.2242	2.5
45–54	66 (24.4)	4 (15.4)	70 (23.6)	0.4673	5.7
55–64	73 (26.9)	8 (30.8)	81 (27.3)	0.6507	9.9
≥ 65	60 (22.1)	5 (19.2)	65 (21.9)	1.0000	7.7
Cause of death					
Cardiac	258 (95.2)	25 (96.2)	283 (95.3)	1.0000	8,8
Noncardiac^++^	13 (4.8)	1 (3.8)	14 (4.7)		7,1
Organized competition or event					
Yes	15 (5.5)	3 (11.5)	18 (6.1)	0.2007	16,7
No	256 (94.5)	23 (88.5)	279 (93.9)		8,2
Activity					
Ice hockey	20 (7.4)	6 (23.1)	26 (8.8)	0.0171^a^	23.1
Swimming	17 (6.3)	3 (11.5)	20 (6.7)	0.3996	15
Physical conditioning	13 (4.8)	3 (11.5)	16 (5.4)	0.1549	18.8
Cycling	59 (21.8)	2 (7.7)	61 (20.5)	0.1253	3.3
Jogging	20 (7.4)	2 (7.7)	22 (7.4)	1.0000	9.1
Off-ice hockey	7 (2.6)	2 (7.7)	9 (3)	0.1812	22.2
Golfing	5 (1.8)	2 (7.7)	7 (2.4)	0.1178	28.6
Hiking and/or taking a walk	20 (7.4)	1 (3.8)	21 (7.1)	1.0000	4.8
Snowmobile	9 (3.3)	1 (3.8)	10 (3.4)	0.6058	10
Basketball	4 (1.5)	1 (3.8)	5 (1.7)	0.3696	20
Hunting	24 (8.9)	0 (0)	24 (8.1)	0.2463	0
Navigation	13 (4.8)	0 (0)	13 (4.4)	0.6135	0
Snowshoeing	10 (3.7)	0 (0)	10 (3.4)	1.0000	0
Alpine skiing	9 (3.3)	0 (0)	9 (3)	1.0000	0
All-terrain vehicles	9 (3.3)	0 (0)	9 (3)	1.0000	0
Cross-country skiing**	6 (2.2)	0 (0)	6 (2.0)	1.0000	0
Other activities	26 (9.6)	3 (11.5)	29 (9.8)	0.7287	10.4*
Total	271 (100)	26 (100)	297 (100)		8.8

Values are numbers (%). Fisher’s exact tests were conducted between the recommendation and non-recommendation groups for categorical variables. The global test *p*-values are as follows: sex (1.0000), age group (0.0337), cause of death (1.0000), organized competition or event (0.2007), and activity (0.0359). The global P-value for activity was estimated using Monte-Carlo simulations. ^++^The noncardiac category includes one death (*n* = 1) of undetermined cause. *The percentage of reports containing at least one recommendation for physical education-related natural deaths is 66.7: two deaths related to undefined activities (each report containing recommendations) and one cross-country skiing-related death (no recommendation) occurred in the context of a physical education class. **Including one case (no recommendation) that occurred in the context of a physical education class. ^a^*p* < 0.05.

Recommendations were found in 8.8% (*n* = 26) of the investigation reports related to natural deaths in sport and recreation. While males represented 92.3% (*n* = 24) of the reports that contained at least one recommendation, the rate of recommendations (proportion of reports containing at least one recommendation, relative to the number of deaths per sex) was similar between males (8.7%) and females (9.1%). Among all age groups, individuals aged 18–24 years (*n* = 13, representing 4.4% of all natural deaths) had the highest rate of recommendations relative to age (30.8%), although most natural deaths were observed in individuals aged 45 and over (72.7%, *n* = 216). Accordingly, a significant difference was observed between the recommendations and non-recommendations group for the age group categories (*p* = 0.0337) for the global test, with individuals aged 18–24 years being over-represented (*p* = 0.0193) in the recommendations group.

Most of the cases involved deaths from cardiac causes (95.3%, *n* = 283) and fatalities occurring in the context of organized competitions or events (93.9%, *n* = 279). However, no statistically significant differences were observed between the recommendations and non-recommendations group in both categories.

For the three activities associated with the highest number of deaths (cycling: 20.5%, *n* = 61; ice hockey: 8.8%, *n* = 26; and hunting: 8.1%, *n* = 24), ice hockey had a relatively high rate of reports with recommendations (23.1%, *n* = 6), whereas cycling had a low rate of recommendations (3.3%), and hunting had no recommendations (0%). A significant difference was observed between the recommendations and non-recommendations group for the activities (*p* = 0.0359) for the global test, with ice hockey being over-represented (*p* = 0.0171) in the recommendations group (Table 1).

### 3.2 Nature of coroners’ recommendations

The 60 recommendations were analyzed individually to determine the presence of the seven components of the model based on public health principles and were further categorized by activity (Table 2). Ice hockey was the activity with the highest number of recommendations, with 35% (*n* = 21) of all instances. Considering all activities, the priority population, the risk/contributing factors [behavior or lifestyle, environmental exposure or inherited characteristic (5)] and the associated countermeasures were identified in all recommendations (100%, *n* = 60). The strategy for implementation (95%, *n* = 57) and the organization responsible to enact the recommendation (93%, *n* = 56) were also identified in most cases (provincial government: *n* = 29; municipalities: *n* = 9; nongovernment organizations: *n* = 9; federal government: *n* = 3, statutory authority: *n* = 3, multiple organizations: *n* = 3) (data not shown). The level of intervention was stipulated in 80% (*n* = 48) of the recommendations: advocacy and sensibilization (41.7%, *n* = 25), policy refinement or development (33.3%, *n* = 20) and legislative development or change (5%, *n* = 3) (data not shown). The time frame was stipulated in 11.7% (*n* = 7: implicitly in all cases, but not explicitly mentioned) of all recommendations.

**Table 2:** Frequency and proportion of components related to the nature of coroner’s recommendations for natural deaths associated with sport and recreation, by activity and for all recommendations in Québec, Canada, from January 1, 2006, to December 31, 2019 (inclusive).

Activity related to the death	Number of recommendations	Proportion of recommendations per 100 activity-related natural deaths	Priority population	Risk/contributing factor	Countermeasure	Level of intervention	Strategy for implementation	Organization responsible to enacting the recommendation	Time frame for implementation
Ice hockey	21	80.8	21 (100)	21 (100)	21 (100)	18 (85.7)	21 (100)	19 (90.5)	6 (28.6)
Swimming	7	35	7 (100)	7 (100)	7 (100)	5 (71.4)	5 (71.4)	7 (100)	1 (14.3)
Jogging	7	31.8	7 (100)	7 (100)	7 (100)	7 (100)	7 (100)	7 (100)	0 (0)
Off-ice hockey	6	66.7	6 (100)	6 (100)	6 (100)	5 (83.3)	6 (100)	6 (100)	0 (0)
Physical conditioning	5	31.3	5 (100)	5 (100)	5 (100)	3 (60)	5 (100)	3 (60)	0 (0)
Physical education (undefined activity)	5	166.7*	5 (100)	5 (100)	5 (100)	3 (60)	5 (100)	5 (100)	0 (0)
Cycling	3	4.9	3 (100)	3 (100)	3 (100)	3 (100)	3 (100)	3 (100)	0 (0)
Golfing	2	28.6	2 (100)	2 (100)	2 (100)	2 (100)	2 (100)	2 (100)	0 (0)
Hiking and/or taking a walk	1	4.8	1 (100)	1 (100)	1 (100)	0 (0)	1 (100)	1 (100)	0 (0)
Combat sports (Kickboxing/Muay Thai)	1	33.3	1 (100)	1 (100)	1 (100)	0 (0)	1 (100)	1 (100)	0 (0)
Snowmobile	1	10	1 (100)	1 (100)	1 (100)	1 (100)	0 (0)	1 (100)	0 (0)
Basketball	1	20	1 (100)	1 (100)	1 (100)	1 (100)	1 (100)	1 (100)	0 (0)
Total	60	20.2	60 (100)	60 (100)	60 (100)	48 (80)	57 (95)	56 (93.3)	7 (11.7)

Values are numbers (%). *This includes two deaths associated with undefined activities (totaling five recommendations) and one death related to cross-country skiing during a physical education class, which did not lead to a recommendation.

Percentage of agreement (excluding the five recommendations analyzed collectively) and inter-rater reliability varied for the seven components of the model: 1—Priority population (92.7%, PABAK = 0.8545), 2—Risk/contributing factor (100%, PABAK = 1), 3—Countermeasure (98.2%, PABAK = 0.9636), 4—Level of intervention (90.9%, PABAK = 0.8182), 5—Strategy for implementation (94.5%, PABAK = 0.8909), 6—Organization responsible to enacting the recommendation (94.5%, PABAK = 0.8909), and 7—Time frame for implementation (98.2%, PABAK = 0.9636).

### 3.3 Thematic analysis of coroners’ recommendations

The thematic analysis revealed that 58.3% (*n* = 49) of the themes covered in the recommendations concerned automated external defibrillators (AED): 1—AEDs in police vehicles (2.4%, *n* = 2), 2—Allowing use of AEDs by the public (7.1%, *n* = 6), 3—AEDs (presence and accessibility) in public or at-risk areas (21.4%, *n* = 18), in addition to the issue of 4—Periodic training: AEDs and Cardiopulmonary Resuscitation (CPR) (27.4%, *n* = 23). Ambulance coverage and pre-hospital care (10.7%, *n* = 9), as well as diagnosis and early management of cardiovascular disease and issues related to the quality of care were also recurring topics (13.1%, *n* = 11) (Table 3).

**Table 3:** Frequency and proportion of themes* covered in coroner’s recommendations for natural deaths associated with sport and recreation, by activity and for all recommendations in Québec, Canada, from January 1, 2006, to December 31, 2019 (inclusive).

Activity related to the death	Number of themes covered	A—AEDs in police vehicles	B—Allowing use of AEDs by the public	C—AEDs (presence and accessibility) in public or at-risk areas	D—Periodic training (AEDs and CPR)	E—Ambulance coverage and pre-hospital care	F—Diagnosis and early management of cardiovascular disease and issues related to the quality of care	G—Developing awareness (reading coroner’s reports or certain recommendations)	H—Development, improvement, distribution or training related to tools, programs, protocols, plans or guides	I—Sports venues and recreation sites: supervision, response procedure, or evacuation capacity, and compliance with standards
Ice hockey	30	1 (3.3)	6 (20)	6 (20)	10 (33.3)	5 (16.7)	0 (0)	2 (6.7)	0 (0)	0 (0)
Off-ice hockey	10	0 (0)	0 (0)	2 (20)	2 (20)	0 (0)	4 (40)	0 (0)	2 (20)	0 (0)
Physical conditioning	10	1 (10)	0 (0)	2 (20)	4 (40)	1 (10)	1 (10)	0 (0)	1 (10)	0 (0)
Swimming	9	0 (0)	0 (0)	2 (22.2)	2 (22.2)	1 (11.1)	1 (11.1)	1 (11.1)	1 (11.1)	1 (11.1)
Jogging	7	0 (0)	0 (0)	1 (14.3)	1 (14.3)	2 (28.6)	1 (14.3)	0 (0)	1 (14.3)	1 (14.3)
Physical education (undefined activity)	5	0 (0)	0 (0)	1 (20)	3 (60)	0 (0)	0 (0)	0 (0)	1 (20)	0 (0)
Golfing	4	0 (0)	0 (0)	2 (50)	0 (0)	0 (0)	0 (0)	2 (50)	0 (0)	0 (0)
Cycling	3	0 (0)	0 (0)	0 (0)	0 (0)	0 (0)	3 (100)	0 (0)	0 (0)	0 (0)
Kickboxing/Muay Thai	2	0 (0)	0 (0)	1 (50)	1 (50)	0 (0)	0 (0)	0 (0)	0 (0)	0 (0)
Snowmobile	2	0 (0)	0 (0)	0 (0)	0 (0)	0 (0)	1 (50)	1 (50)	0 (0)	0 (0)
Hiking and taking a walk	1	0 (0)	0 (0)	0 (0)	0 (0)	0 (0)	0 (0)	0 (0)	0 (0)	1 (100)
Basketball	1	0 (0)	0 (0)	1 (100)	0 (0)	0 (0)	0 (0)	0 (0)	0 (0)	0 (0)
Total	84	2 (2.4)	6 (7.1)	18 (21.4)	23 (27.4)	9 (10.7)	11 (13.1)	6 (7.1)	6 (7.1)	3 (3.6)

Numbers are values (%), AED, automated external defibrillators; CPR, cardiopulmonary resuscitation. *For examples of recommendations for each theme listed in the table, please refer to Supplemental material.

Percentage of agreement and inter-rater reliability (excluding the five instances analyzed collectively) varied between themes (as lettered in Table 3): A—(100%, PABAK = 1), B—(100%, PABAK = 1), C—(98.2%, PABAK = 0.9636), D—(100%, PABAK = 1), E—(100%, PABAK = 1), F—(94.5%, PABAK = 0.8909), G—(100%, PABAK = 1), H—(98.2%, PABAK = 0.9636), I—(98.2%, PABAK = 0.9636).

## 4 Discussion

This study provided a holistic investigation into coronial recommendations regarding natural deaths in the context of sport and recreation. It encompasses the analysis of demographic and contextual specifics of cases, the nature of recommendations aligning with public health principles, as well as the recurring themes identified by coroners.

### 4.1 Frequency and rate of coroners’ recommendations

Coroners’ recommendations were present in 8.8% of the investigation reports of deaths associated with sport and recreation, for the period between January 2006 and December 2019, and the number of reports containing recommendations decreased over the analyzed period. The fact that AEDs accounted for a large proportion of the themes covered in the recommendations and that this device was progressively more present in the province of Québec within the analyzed period can contribute to explaining both the rare occurrence of coroners’ recommendations and the downward trend observed.

Of the reports containing at least one recommendation, 92.3% were related to males. Nevertheless, the rate of recommendations was similar between sexes, possibly due to the universality of the prevention strategies suggested by the coroners, primarily centered on the issue of AEDs. In addition, recommendations were more likely to be found in reports related to persons aged 18–24 years. This is noteworthy, as those aged 45 and over were the ones who were dying more frequently. The highly emotive subject of death in children and young adults, and the potential for these cases to be more mediatized was hypothesized to explain similar results (5). However, individuals aged 45 and over warrant close attention since they are more likely to die of sudden cardiac death than their younger counterparts (22–24). Recommendations were also more likely to be found in reports for deaths associated with ice hockey. On the other hand, cycling and hunting activities displayed a high frequency of deaths but a low proportion of coroners’ recommendations per incident. Given that cycling and hunting are two of the three primary activities associated with natural deaths in sport and recreation in the province of Québec, this finding calls for a more comprehensive investigation. While recommendations related to AEDs for hockey arenas may be relatively easier to implement, addressing the issue of natural deaths in activities practiced in more remote areas, such as cycling and hunting, will require creative efforts to be taken care of.

### 4.2 Nature of coroners’ recommendations

When considering all activities, six out of seven components related to the nature of recommendations were present in at least 80% of the recommendations, confirming the coroners’ reliance on public health principles in Québec. The time frame, however, was specified in only 11.7% of the recommendations. It is important to note that, during the analyzed period, recipients of recommendations in Québec were neither legally obligated to implement the coronial recommendations (12) nor required to respond to them (mandatory since November 2022) (10). This lack of obligation may explain the infrequent inclusion of time frames in the recommendations. These findings align with that of other investigations (5, 6) where it has been emphasized that specifying a clear time frame in a recommendation appears crucial because vague deadlines typically do not encourage a shift in behavior (6). It is worth acknowledging, however, that AEDs were increasingly implemented in sport and recreation facilities in Québec (especially in arenas) during the analyzed period.

In the realm of preventive interventions, those that involve modifying an individual’s physical environment are typically categorized as passive measures. In contrast, active measures require individuals to actively participate in safeguarding themselves. While these terms are not entirely dichotomous, more passive approaches, such as environmental changes that demand minimal human involvement, are often considered highly effective (5). In the current study, most countermeasures involved AED implementation, delivery, or training. Therefore, a holistic approach that spans a broad range of the passive-active spectrum may prove to be optimal. For instance, raising awareness and advocating for public engagement could complement a legislation aiming at maximizing the implementation, delivery and training associated with AEDs.

### 4.3 Thematic analysis of coroners’ recommendations

Out-of-hospital cardiac arrest (OHCA) is a significant public health concern in Canada (22). Early bystander CPR and prompt defibrillation are critical determinants for improving survival rates in cases of sudden cardiac arrest (25). In the current investigation, deaths attributed to cardiac causes accounted for 95.3% of the total burden. It is therefore unsurprising that almost 60% of the themes covered in the coroner’s recommendation in this study were related to AED implementation, delivery and training. CPR and timely defibrillation were moreover elsewhere suggested to be linked to higher survival rates among exercise-related OHCA cases compared to non-exercise related events (25). This is likely because some sport and recreation venues are equipped with AEDs, given that survival decreases by 7–10% every minute without effective treatment of lethal arrhythmias (26, 27).

In many cases, however, AEDs availability may be an issue. On the one hand, although most police vehicles in Québec are equipped with AEDs as of 2024, accessibility can be very challenging for first responders in remote or rural areas (28). On the other hand, the provincial critical emergency response time targets set in 2000 (90% of responses within 8 min in urban areas, 12 min for suburban areas, and 30 min for rural areas), as outlined in the report of the Comité national sur la révision des services préhospitaliers d’urgence (commonly known as the *Rapport Dicaire*) (29), are likely too lengthy for the effective management of lethal arrhythmias (26, 27). Notably, these emergency response time targets are still not consistently met as of 2024, as outlined in several mediatized cases. In this context, efforts to ensure the strategic deployment and efficient delivery of AEDs throughout the province of Québec appear crucial (30). This is especially true in rural areas, where longer response times are an issue (28). Noteworthy, four out of the five activities associated with the highest number of natural deaths in sport and recreation (cycling, hunting, jogging, hiking or taking a walk, *n* = 128, 43.1% of all deaths) during the studied period are prone to occur in places lacking AEDs (roads, cycling paths, hunting territories and outdoor sites). Therefore, innovative perspectives like an optimized drone network designed to reduce delivery time of AEDs in case of an OHCA deserve consideration (31, 32). In this context, the innovative multi-faceted approach to improve public-access defibrillation (33) that was developed by the International Liaison Committee on Resuscitation in 2022 warrants serious scrutiny, as it has the potential to guide integrated and evidence-based decision-making in the province of Québec.

The theme of “*Diagnosis and early management of cardiovascular disease and care quality issues*” was the third most frequently recorded in our study. While familial predisposition, particularly to sudden cardiac death, is a significant documented factor to be considered in a prophylactic approach aimed at preventing natural deaths associated with sports and recreation (34–37), the coroners did not directly address this topic in the recommendations examined in our study. It is important to note, however, that during medical screening, patients are typically asked minimally about family medical history and potential hereditary diseases. Physicians can also assess the need for clinical and genetic evaluations (38, 39). Moreover, while hereditary factors may not be explicitly addressed in coroners’ recommendations in our study, they are systematically considered and discussed by coroners who directly communicate with the family of the deceased to investigate the causes of death. In this context, the clinical and genetic evaluation of surviving potentially at-risk family members (38, 39) can be assessed by the patient’s physician. Besides, only three recommendations addressed premedical screening, which is lower than expected. Sudden cardiac death ranks as the primary cause of non-traumatic fatalities in athletes (40, 41). Although rare, these deaths can result from identifiable hereditary or congenital heart conditions that can be addressed with preventive screening strategies (9, 40, 42, 43). It must be emphasized, however, that almost all of the deaths in the current study do not involve high-level athletes but rather regular sportsmen and sportswomen. Hence, proactive and systematic screening for cardiovascular risk factors appears crucial, particularly for individuals aged ≥35 years, who accounted for 86.2% (*n* = 256) (Table 1) of all natural deaths in sport and recreation over a 14-year period and are most likely to succumb to cardiac infarction (23, 41, 44–48).

In 2013, the Canadian government established a program that equipped all hockey arenas in Canada with an AED, which explains why all six occurrences of this theme that were associated with ice hockey were observed in 2013 or earlier. Devices were also progressively installed in fitness centers, including gyms and public pools. However, this installation was not uniform across the province since there is no legislation in force as of 2024. Québec ranked second to last in terms of AED availability, with just 27 AEDs per 100,000 inhabitants, according to a Canadian survey conducted between February 2019 and June 2020 that analyzed data from various Canadian provinces and cities (27). In contrast, Manitoba leads the provinces surveyed with a substantial 324 AEDs per 100,000 inhabitants. This notable difference can be traced back to Manitoba’s legislation (*Defibrillator Public Access Act*, in force in 2013) (49) which mandates AED placement in high-traffic public places such as gyms, arenas, community centers, golf courses, schools and airports. In June 2020, the Ontario government followed suit by enacting a similar legislation (50). Considering the example of these provinces, adopting similar legislation in Québec to systematically enhance access to AEDs could warrant serious consideration, as it has the potential to save more lives. Noteworthy, implementing such legislation has been linked to higher rates of CPR and public AED use, along with improved survival rates for patients experiencing out-of-hospital cardiac arrest (51).

To prevent natural deaths in sport and recreation, schools are strategic locations to target. Gymnasiums and sports venues in schools in Québec are not only used by students for physical education classes and sports, but they are also used by adults outside regular school hours. Since September 2019, as part of the initiative of the Advanced Coronary Treatment Foundation, all secondary schools in the province of Québec have been equipped with an AED (52). Elementary schools, however, are not required to be equipped with this device (52), and AEDs are hence not yet universally present in these institutions.

It is noteworthy, however, that nearly all elementary schools in the Québec City area surveyed in December 2018 possessed an AED (52), and the primary barriers to its effective utilization were found to be the lack of AED training and the fear of using the device. Based on this knowledge, the introduction of an intervention to professionals in elementary schools using a concise educational video resulted in a 29% reduction in the time taken to administer the first shock during a pediatric sudden cardiac arrest simulation (52). Undoubtedly, advocating for both AED access and training is crucial to improve outcomes in cases of sudden cardiac arrest within schools (53), and likely applies to other sport and recreation venues as well. Since 2017, CPR training has been embedded within the academic curriculum and became mandatory for secondary school students (Secondary III, ~14 years old) in the province of Québec (52). This decision aligns with the trend seen in several countries and states since 2003, where legislation mandates CPR and AED education (54, 55). However, the mere presence of legislation or inclusion in the curriculum does not guarantee effective implementation (55). To ensure success, it is crucial to provide support for implementation and to establish a diligent monitoring system (55). Furthermore, in Québec’s education system, childcare workers are the only ones required to complete a general first aid course, while school staff and support staff have the option to undergo such training voluntarily (52). Therefore, the objective to equip all schools in Québec (elementary and secondary schools) with an AED is important, and a systematic and supportive approach to proper training in school settings should be carefully considered to overcome the main obstacles in AED usage. Lastly, considering that this study found periodic training (AED and CPR) to be the most recurring theme (considering all activities and associated sport venues), a holistic approach focusing on AED access and training would likely benefit not only schools but also all sport and recreation practice sites. Mandatory training in elementary and secondary schools would assure that students who finished secondary school will have been trained and certified at least twice.

## 5 Strengths, limitations, and perspectives

Given that this study is built upon prior research, some strengths and limitations have been elucidated in a previous publication (4). Prominent strengths include the thoroughness of the CD-BCQ database, the rigorousness of the data collection process, the utilization of two distinct denominators (56), and the meticulousness of the data entry procedures. These procedures were tailored to match the at-risk population within the ÉBARS, ensuring precise and congruent numerator values for participation-based rates (4). However, it is important to acknowledge certain limitations. Firstly, there is the possibility that the BCQ algorithms might have failed to identify some cases, especially those associated with emerging activities. Additionally, some natural deaths in sport and recreation and associated coronial recommendations may not have been detected for cases still under investigation. This effect is however most likely minimal since the percentage of completed coroner investigations (all coronial investigations in the province) for 2019 was 96.2%, while the 2006–2018 period was considered closed (100%) when data were extracted (4). In addition, this study did not consider variations in exposure frequency within the participation data, and the presence of gaps in the years covered by participation data might have had an impact on the accuracy of participation-based rates. Furthermore, it is noteworthy that specific activities, age groups and populations were not encompassed in the ÉBARS surveys. When analyzing the nature of coroners’ recommendations, a separate examination of each element was conducted in accordance with previous investigations (5, 6). However, the underlying theoretical principles of the model used implies that some elements would overlap and influence each other (5). While the flexibility of thematic analysis is a notable advantage of this method, it is worth noting that the same dataset can yield a wide range of potential interpretations, which could be considered a potential drawback (13). However, it is important to emphasize that each steps of the methods employed in this study were meticulously followed by the investigators (13, 14), and the narrative and interpretations derived from the data were validated by a board-certified cardiologist. Moreover, although the number of reports containing recommendations included in our analysis was small and required careful consideration in the statistical analysis (6, 21), it represents all available data on this issue over a 14-year period, within the limitations of our data extraction and treatment procedures (4). The investigation of coroners’ recommendations, which are rarely studied regardless of context (1, 5–7), within the limited research area of natural death in sport and recreation (8, 9, 24, 43, 57, 58), offers valuable insights into this important public health issue. Besides, the coroners’ reports used in the study did not consistently provide detailed causes of death (e.g., hypertrophic cardiomyopathy, Brugada syndrome, aortic rupture), leading to the use of broad categories (cardiac and noncardiac). This lack of detailed delineation is a limitation of the study, as more precise information could have provided valuable prevention insights, given that medical approaches are tailored to specific conditions. Additionally, analyzing recommendations in relation to more specific causes of death could have provided deeper insights into why certain cases received recommendations while others did not. However, the focus on coroners’ recommendations as presented in the reports (which are intended for recipients who are expected to implement preventive measures) highlights an occasional lack of detail, pointing to an area for improvement in how coroners document cases to better support recommendations. Furthermore, the potential reasons for variations in the presence or absence of recommendations across different activities may be attributed to factors such as age and specific causes of death. Hence, to gain a comprehensive understanding of natural deaths in sport and recreation, the etiology of fatalities based on autopsy data will be investigated further in a separate study. Lastly, for a holistic and complete investigation of all deaths associated with sport and recreation in Québec, a specific investigation of the coroner’s recommendations related to unintentional injury deaths is also necessary.

## 6 Conclusion

This study examined the frequency, the nature, and the themes of coronial recommendations associated with natural deaths in sport and recreation for the period running from January 2006 to December 2019. Investigation reports of individuals aged 18–24 years or related to ice hockey were significantly more likely to contain recommendations. However, the study shed light on concerning trends, with cycling and hunting activities, along with individuals aged 45 and over, demonstrating a higher frequency of deaths and a relatively lower rate of recommendations. Addressing the heightened risk of sudden cardiac arrest in those aged 45 and over, as well as the challenge of timely treatment for lethal arrhythmias in remote areas, emerge as priorities. Moreover, while six out of the seven components of the public health principles model used in this study were found in at least 80% of the recommendations, time frame for implementation was rarely stipulate, which represent a potential avenue for improvement. Nearly 60% of the themes addressed in the coroner’s recommendations pertained to AED implementation, delivery and training. The innovative multi-faceted approach to improving public-access defibrillation (33) is a holistic and thorough approach that effectively addresses these three critical topics, with the potential to inform comprehensive and evidence-based decision-making in Québec. Finally, the coroners’ reports used in the study did not consistently provide detailed causes of death, leading to broad categorizations of causes of deaths, which may have resulted in lost prevention insights; this limitation underscores the need for investigating the etiology of fatalities based on autopsy data.

## Author’s note

During the preparation of this work, the authors used OpenAI’s ChatGP to correct and enhance the text for improved language quality. After using this tool, the authors reviewed and edited the content as needed and take full responsibility for the content of the publication. The text was subsequently revised by the linguistic services of the Ministère de l’Éducation du Québec.

## Data availability statement

The raw data supporting the conclusions of this article will be made available by the authors, without undue reservation. All data used were obtained from the computerized database of the Bureau du coroner du Québec. Requests for data use may be sent to the Bureau.

## Ethics statement

Ethical approval was not required for the study involving humans in accordance with the local legislation and institutional requirements. Written informed consent to participate in this study was not required from the participants or the participants’ legal guardians/next of kin in accordance with the national legislation and the institutional requirements. This is a retrospective descriptive study based on de-identified data of deceased individuals. The authors confirm that patient consent is not applicable to this article.

## Author contributions

PR: Conceptualization, Data curation, Formal analysis, Investigation, Methodology, Project administration, Supervision, Validation, Writing – original draft, Writing – review & editing. P-AP: Data curation, Investigation, Methodology, Writing – original draft, Writing – review & editing. JS-M: Formal analysis, Methodology, Software, Writing – original draft, Writing – review & editing. PP: Investigation, Methodology, Validation, Writing – original draft, Writing – review & editing.
