# Supplementary material for: Insights from coronial recommendations for preventing natural deaths in sport and recreation in Québec, Canada
Source: Front Public Health. 2024 Jul 31;12:1389675. doi: 10.3389/fpubh.2024.1389675 (PMC11323781; doi:10.3389/fpubh.2024.1389675)
Supplement: Supplementary file 1 [file Data_Sheet_1.docx]

Supplementary Material

Insights from Coronial Recommendations for Preventing Natural Deaths in Sport and Recreation in Québec, Canada

Philippe Richard^1*^, Paul-André Perron^2^, Jérémie Sylvain-Morneau^3^, Paul Poirier^4,5^

^1^ Direction de la sécurité dans le loisir et le sport, Ministère de l’Éducation, Québec, QC, Canada

^2^ Bureau du coroner du Québec, Québec, QC, Canada

^3^ Institut national de santé publique du Québec, Québec, QC, Canada

^4^ Institut universitaire de cardiologie et de pneumologie de Québec, Université Laval, Québec, QC, Canada

^5^ Faculty of Pharmacy, Université Laval, Québec, QC, Canada

*** Correspondence:**Philippe Richard
philippe.richard@education.gouv.qc.ca

Examples of Recommendations (2006-2019) for Each Theme Listed in Table 3 (A to I)

**A – AEDs in police vehicles**

*To the City of xxx:*

*To equip all public safety patrol vehicles with automated external defibrillators.*

**B – Allowing use of AEDs by the public**

To the government of xxx:

*To adopt a regulation allowing the use of automated external defibrillators by the public, thereby repealing the exclusive use reserved for physicians*.

**C – AEDs (presence and accessibility) in public or at-risk areas**

*To the City of xxx****:***

*In its criteria for granting or renewing permits for a sports center, to ensure that the establishment is equipped with an automated external defibrillator.*

**D – Periodic training (AEDs and CPR)**

*To the City of xxx****:***

*In its criteria for granting or renewing permits for a sports center, to ensure that the personnel are trained in cardiopulmonary resuscitation, including the correct use of an automated external defibrillator.*

**E – Ambulance coverage and prehospital care**

*To the Regional Health and Social Services Agency of xxx:*

*To ensure the establishment of an ambulance dispatch center that will use a recognized call classification system and will be able to inform regional or provincial authorities about response time delays within various call categories.*

**F – Diagnosis and early management of cardiovascular disease and issues related to the quality of care**

*To the networks of Federation of xxx:*

*To emphasize the importance and necessity of having a health assessment before engaging in physical activities that require sustained physical effort, in their advertisements inviting seniors to participate in such activities.*

**G – Developing awareness (reading coroners’ reports or certain recommendations)**

*To distribute this investigation report to all Health and Social Services Agencies in the province.*

**H – Development, improvement, distribution or training related to tools, programs, protocols, plans or guides**

*To the Ministry of xxx:*

*To develop a specific guide for medical emergencies during road running events and ensure its dissemination to organizers of such activities.*

**I – Sports venues and recreation sites: supervision, response procedure, or evacuation capacity, and compliance with standards**

*To the City of xxx:*

*To ensure that, when a sporting event takes place within its territory, the medical and organizational infrastructure meets the established standards, and to refrain from authorizing the event if these standards are not met.*
